# Case report: A case of severe acute hepatitis of unknown origin

**DOI:** 10.3389/fped.2022.975628

**Published:** 2022-10-05

**Authors:** Yu-Jiao Zhou, Hui-Ying Gu, Qi-Qin Tang, Fan Li, Jin Zhu, Ting Ai, Kun Zhu, Bin-Yue Xu, Qing Wang, Ai-Long Huang, Juan Chen, Zhen-Zhen Zhang

**Affiliations:** ^1^The Key Laboratory of Molecular Biology of Infectious Diseases Designated by the Chinese Ministry of Education, Chongqing Medical University, Chongqing, China; ^2^Ministry of Education Key Laboratory of Child Development and Disorders, Chongqing Key Laboratory of Child Infection and Immunity, Department of Infectious Disease, National Clinical Research Center for Child Health and Disorders, Children’s Hospital of Chongqing Medical University, Chongqing, China; ^3^Department of Endocrine and Breast Surgery, The First Affiliated Hospital of Chongqing Medical University, Chongqing, China; ^4^Department of Pathology, Children’s Hospital of Chongqing Medical University, Chongqing, China; ^5^Department of Radiology, Children’s Hospital of Chongqing Medical University, Chongqing, China; ^6^Chongqing Municipal Center for Disease Control and Prevention, Chongqing, China; ^7^Key Laboratory of Laboratory Medical Diagnostics, Chinese Ministry of Education, Chongqing Medical University, Chongqing, China

**Keywords:** case report, unexplained acute hepatitis, torque teno virus, immune injury, methylprednisolone

## Abstract

According to analyses of etiology, clinical features, diagnostic methods, and treatment strategies by summarizing a case of unexplained acute hepatitis recently experienced, we are aiming to provide some information to enrich the clinical experience in diagnosis and treatment of severe acute hepatitis of unknown etiology in young children. A boy, aged 10 years and 6 months old, was admitted to the hospital due to acute abdominal pain, jaundice, and exceptionally high levels of ALT and AST. A range of measures, including patient history, physical examination, and routine laboratory testing, were performed. Furthermore, strategies such as trio-based next-generation sequencing (Trio-NGS) and liver biopsy, as well as metagenomic NGS (mNGS) of blood and liver samples were also performed. In summary, this case was an acute severe non-A–E hepatitis that is a probable case with hepatitis of unknown origin. Immunohistochemical analysis showed an immune injury in liver tissues. Torque teno virus (TTV) sequences were detected by mNGS assay. As for treatment strategies, in addition to general treatment, this patient also underwent plasmapheresis and methylprednisolone treatment due to disease deterioration. The patient’s liver function was improved afterward and discharged after one month of treatment. Taken together, this work reported the clinical feature and treatment of severe acute hepatitis with non-A–E hepatitis in detail. The potential mechanism of liver damage might be due to an immune attack in which TTV might play a role as a co-factor.

## Introduction

Liver disease of unknown etiology refers to liver diseases that cannot be clearly diagnosed by patient history, physical examination, and routine laboratory testing. Due to differences in race and region, the reported incidence of unexplained liver disease in pediatric patients is variable, ranging from 10 to 50% (1–4). The etiologies of unexplained liver disease can be roughly divided into infectious and non-infectious categories. Although the common viruses (hepatitis viruses A, B, C, D, and E) that cause acute viral hepatitis are undetectable, infectious etiologies, including Epstein–Barr virus (EBV), cytomegalovirus (CMV), herpes simplex viruses (HSV), bacteria, fungi, and parasites, are still common causes of liver injury (5–9). In addition, non-infectious etiologies, such as non-alcoholic fatty liver disease (NAFLD), drug-induced liver injury (DILI), autoimmune hepatitis (AIH), and inherited metabolic liver disease, also account for a relatively large proportion of hepatitis cases (10–13).

Since the first few cases of severe acute hepatitis of unknown origin were reported among young children in Scotland in March (14), increasing numbers of cases have been reported worldwide. As of 26 May 2022, at least 650 cases of unexplained hepatitis in children ranging from ages 1 month to 16 years have been reported in 33 countries, including England, Spain, Israel, the United States, and Denmark (15). The clinical manifestation is acute hepatic dysfunction with significantly elevated aminotransferase levels. Most of the affected children have jaundice, abdominal pain, nausea, vomiting, and diarrhea but no fever. Thirty-eight patients had received liver transplantation, and at least nine deaths were reported (15). According to the World Health Organization (WHO), the case definition of *Confirmed* is N/A at present. The case definition of *Probable* is a person presenting with an acute hepatitis (non-hepA–E*) with serum transaminase > 500 IU/L (AST or ALT), who is 16 years and younger, since 1 October 2021 (15).

The cause of the pediatric liver disease has not been revealed yet. However, a leading hypothesis is that an infectious agent is the underlying cause or a risk factor. Given the presence in about three-fourths of the investigated cases, adenovirus type 41 was initially suspected to be the causative pathogen (16, 17). Nevertheless, adenovirus does not fully explain the increased severity of the cases. In addition, adenovirus is a seasonally transmitted virus with a peak period of infection from February to April. It can cause severe infection in multiple organs, including the liver, in immunocompromised children (18); however, it is rarely able to lead to severe infection in immunocompetent children (19, 20). Thus, in the updated technical note released by WHO, adenovirus positivity was considered more likely to be a coincidental factor (21).

Our team recently experienced a case with severe acute non-A–E hepatitis. Here, we supplement a few noteworthy points based on this case, hoping to provide more information for pediatricians about the current hepatitis of unknown origin in children.

## Case description

A boy who was 10 years and 6 months old was admitted to the hospital due to abdominal pain that persisted for 4 days. The patient also vomited three times without concomitant fever or diarrhea. He was found to have jaundice when referred to a local hospital. The biochemical tests showed exceptionally high levels of alanine aminotransferase [(ALT) 2330 U/L], aspartate aminotransferase [(AST) 1326 U/L], and total bilirubin [(TB) 74.2 μmol/L]. The patient received no intervention or treatment before being transferred to our hospital. He was previously in good health, denied taking any medications or having a history of exposure to patients with COVID-19, and received the second dose of inactivated COVID-19 vaccine 3 months before admission. At admission, the physical examination revealed that the child had scleral icterus, jaundice, hepatosplenomegaly, upper abdominal tenderness, and a positive Murphy’s sign.

**FIGURE 1:**
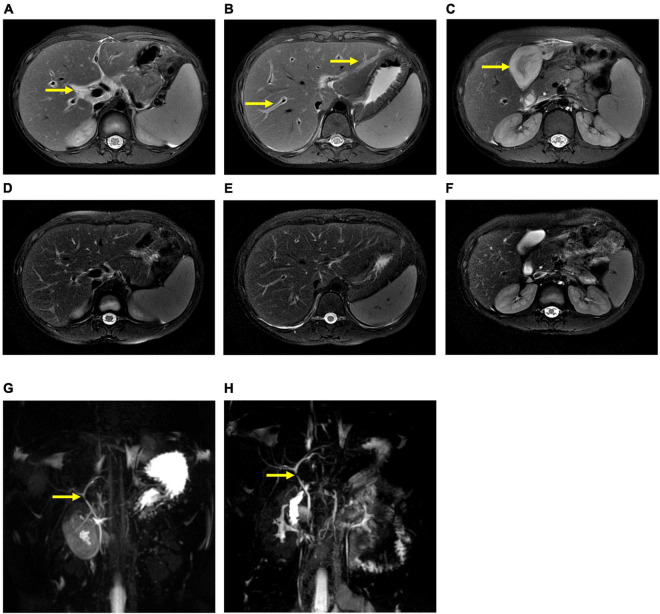
Magnetic resonance cholangiopancreatography (MRCP) results of the patient. MRCP showed edema of the first porta hepatis and the periportal region **(A,B)** and thickness of the gallbladder wall **(C)** at the beginning of treatment (thick arrow), and they were resolved after treatment **(D–F)**. However, MRCP showed a narrow bile duct that was not relieved during hospitalization **(G,H)**.

**FIGURE 2:**
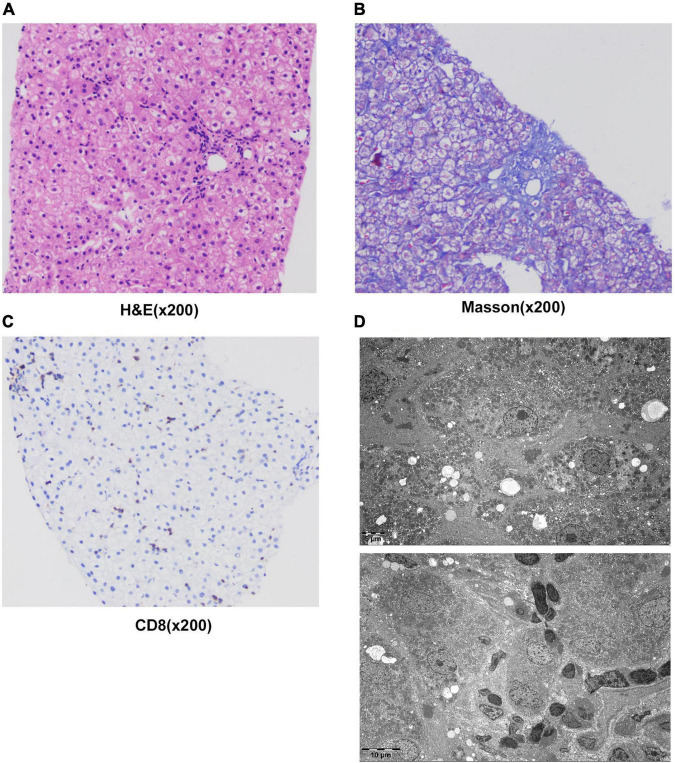
Results of liver histology of the patient. **(A–C)** Liver biopsy results. H&E staining of liver tissue showed swollen hepatocytes and inflammatory cells infiltrating into portal duct areas, and there was mild interfacial inflammation **(A)**. Masson staining indicated mild hepatic fibrosis in the portal area **(B)**. Immunohistochemical staining of CD8 + T-lymphocytes **(C)**. **(D)** The result of electron microscopy showed chronic active hepatitis. No viral inclusions were found.

**FIGURE 3:**
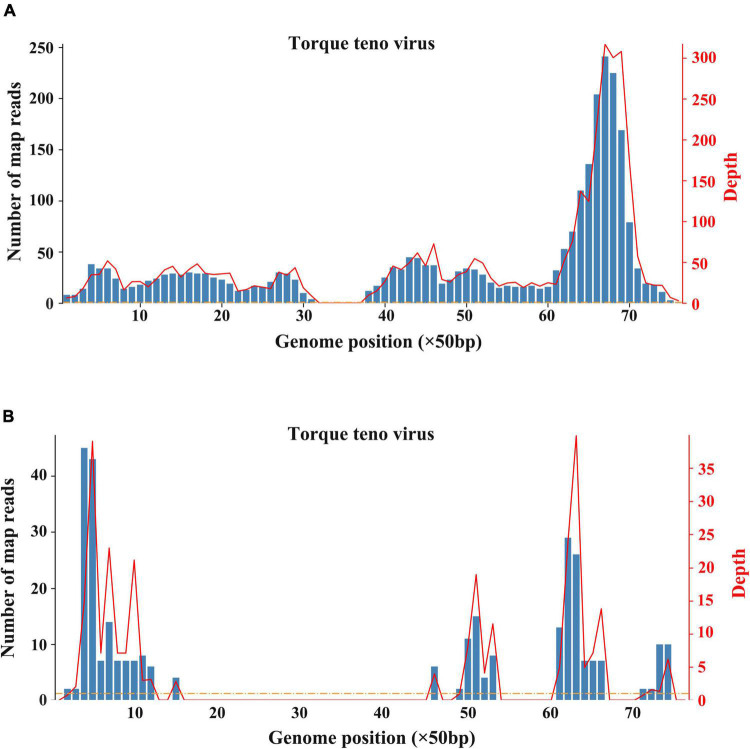
Results of metagenomic sequencing. **(A)** Sequencing results of whole blood. **(B)** Sequencing results of liver tissue.

The patient underwent infectious pathogen screening. Evidence of hepatitis A, B, C, and E viruses, CMV, HSV, and EBV infection is not found by serology (Hepatitis A, B, C, and E virus, CMV, and EBV), immunohistochemical (EBV), and PCR (HSV and EBV) analyses. The autoimmune hepatitis-related antibodies (17 common antibodies including ANA, SSA, AMA M2, and ss-DNA) and metabolism disease-related tests (including blood glucose, blood ammonia, lactate, alpha-fetoprotein, and ceruloplasmin) were also negative. Abdominal ultrasound indicated hepatosplenomegaly, pericholecystic edema, and thickening of the gallbladder wall. Magnetic resonance cholangiopancreatography (MRCP) showed edema of the first porta hepatis, the periportal region, and the gallbladder, as well as stenosis of the choledoch (Figures 1A–C,G). To further clarify the potential cause, the patient underwent trio-based next-generation sequencing (Trio-NGS) and liver biopsy. Trio-NGS did not detect any abnormalities, while liver biopsy showed mild interfacial hepatitis and fibrosis of portal area (G1S1) (Figures 2A,B), and further classification of the intrahepatic lymphocytes predominantly suggested CD8 + cells (Figure 2C). Tests for HBsAg, HBcAg, CMV, IgG4, and EBER in liver tissue remained negative. Although some interlobular bile ducts were very small, the number of intrahepatic bile ducts was normal. Finally, torque teno virus (TTV) sequences were detected by metagenomic NGS (mNGS) in whole blood sample (Figure 3A) and liver tissue (Figure 3B). The result of electron microscopy (Figure 2D) indicated chronic active hepatitis, which was shown as follows: hepatic cells were swollen, rough endoplasmic reticulum and smooth endoplasmic reticulum were slightly expanded, and lipid droplets were observed in hepatocytes (upper image), as well as lymphocytosis were observed in hepatic sinusoidal (lower image). However, no viral inclusions were found.

**FIGURE 4:**
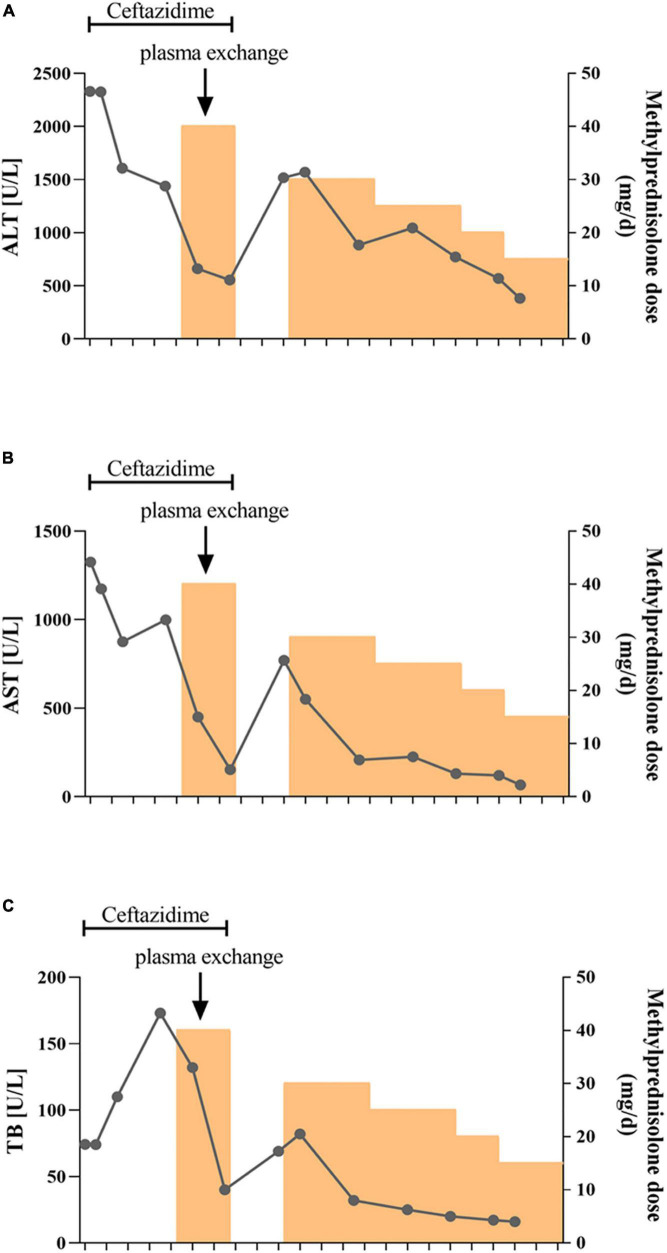
Schematic diagram of the transaminase and bilirubin fluctuations during treatment. The alanine aminotransferase **(A)**, aspartate aminotransferase **(B)**, and total bilirubin **(C)** levels were tested every 3–5 days during treatment. Ceftazidime was used for the first two weeks followed by methylprednisolone treatment. The patient also received plasma exchange once due to rapid aggravation of jaundice.

After hospitalization, the patient received ceftazidime (based on cholecystitis), lipid-soluble vitamins, and reduced glutathione treatment. Unfortunately, the patient’s situation deteriorated with rapid progressive jaundice (peak TB: 173 μmol/L), underwent plasmapheresis once, and was administered methylprednisolone. The patient’s liver function was improved after the administration of steroids (Figures 4A–C). MRCP tests showed that the narrow bile duct was not relieved, but the hepatosplenomegaly and gallbladder edema had disappeared (Figures 1D–F,H). The patient was discharged after one month of treatment. Two weeks post-discharge, he revisited the hospital. He was clinically stable without any adverse and unanticipated events. The biochemical tests showed that ALT was 163 U/L, AST was 60 U/L, and TB was 11.6 μmol/L.

As a whole, this case report follows the CARE Case Report Guidelines. Our adherence to these reporting guidelines has been listed in the Supplementary material.

## Discussion

Patient in this case report presented with gastrointestinal symptoms of abdominal pain and vomiting, elevated transaminases, and jaundice at the onset. He was healthy before disease onset and had no history of taking certain drugs or exposure to poisons. The results of viral hepatitis serology screen were negative, indicating that this case was consistent with the definition of a *Probable* case by the WHO (15).

Viral hepatitis due to hepatitis viruses or to occasionally hepatotropic viruses is one of the main etiological groups of acute or chronic hepatitis in children (22). Recently, with the popularity of hepatitis virus vaccines and the improved detection methods, non-hepatotropic virus (including CMV, EBV, and coxsackievirus) infection-induced acute hepatitis in young children has gained increased attention (23). In the recent outbreak of acute and severe hepatitis of unknown etiology in children, some clues suggested that adenovirus and SARS-CoV-2 might be the etiologies (24). However, until now, there is still a lack of definite evidence on associated mechanisms or causative relationships. As for this case we reported, evidence of adenovirus and SARS-CoV-2 infection was negative, indicating that such two virus infections are unlikely. However, both blood sample and liver tissues were tested positive for torque teno virus (TTV) according to mNGS analysis. TTV is a small single-stranded DNA virus that was discovered in the late 20th century. TTV has an extremely high prevalence worldwide, which is frequently detectable in healthy infants, healthy adults, patients with HBV/HCV, and cases of hepatitis without an obvious viral agent (25–27). Previous studies indicate that TTV is hepatotropic, and TTV infection-induced liver damage could present a diverse spectrum of pathological damage, including ballooning, acidophilia degeneration, formation of apoptosis bodies and focus of necrosis, and mild inflammation in the lobule and portal area (28). Nevertheless, there was no significant difference of TTV DNA positivity in patients with hepatitis when compared to that in healthy controls (28). Moreover, due to the lack of reliable cell culture and animal models, the pathogenicity of TTV remains controversial (27). Notably, most studies assumed that TTV is non-pathogenic. A published article by Okamura et al. reported that genotype 1a of TTV might play a role in the pathogenesis of fulminate hepatitis and chronic liver disease in children liver disease of unknown etiology (29), indicating that some specific genotype of TTV may be pathogenic in children. In the current case, TTV was monitored by mNGS, and its expression level is not high enough to identify the genotype. In addition, no TTV virus particles were observed in liver tissues by electron microscopy. Therefore, before well-grounded evidence emerges, we cannot determine the pathogenicity of TTV. On the contrary, the immunohistochemical analysis showed that IgG4 staining was negative, but the majority of infiltrating inflammatory cells were CD8 + lymphocytes. More importantly, the response of this patient to hormones treatment was good, implying that it is more likely to be an immune injury. Given the uncertainty about the pathogenicity of TTV, we consider that TTV is more likely a co-factor responsible for the inappropriate immune response.

Besides infectious factors, given the immune-mediated hepatic damage, as well as the well-response to methylprednisolone treatment, AIH could not be ruled out yet in this case report. Even though majority of common autoimmune hepatitis-related antibodies were negative, we might also have to consider the possibility of autoantibody-negative autoimmune hepatitis. It has been suggested that seronegative AIH accounts for less than 5% of all adult patients with AIH (30). However, little information is available in children. A retrospective study conducted by Islek et al. found that seven of 54 patients with AIH under 18 years of age were seronegativity persisted during treatment (31), indicating that seronegative AIH could not be ignored in clinical practice. As for this child in our report, he has no documented history of other autoimmune diseases and no typical histologic features of AIH. We considered that the mild interfacial hepatitis is not enough to explain his severe liver damage. Thus, before more substantial evidence emerges, seronegative autoimmune hepatitis cannot be determined.

There were some limitations in the exploration of etiologies in this study. First, the depth and breadth of laboratory testing are not comprehensive enough. Some investigations such as multiplex PCR for respiratory viruses, multiplex PCR gastrointestinal viruses panel, and stool culture for common bacterial enteropathogens are not performed, which might cause the loss of some clinical data. Second, the detection of pathogens and histology was performed after the condition was stable, which is not conducive to the search for etiology. Similarly, no typical manifestations of the acute phase were observed in liver biopsy, which might influence the clinical assessment. Third, this case is a single case report. Continuous follow-up is required to further clarify the clinical characteristics and etiology of such liver diseases in young children.

## Conclusion

Collectively, we report a case with severe acute hepatitis of unknown origin. Based on laboratory examinations and treatment response, we suspect the etiology of this case may be due to an immune injury in which TTV might play a role as a co-factor. We suggest liver biopsy for patients with severe acute hepatitis of unknown origin and trial steroid therapy when the liver damage is similar to autoimmune hepatitis. By reporting this case, we expect to add further support to the notion that immune dysfunction might be the main cause of liver damage in children with acute hepatitis of unknown origin.

## Data availability statement

The datasets for this article are not publicly available due to concerns regarding participant/patient anonymity. Requests to access the datasets should be directed to the corresponding authors.

## Ethics statement

The studies involving human participants were reviewed and approved by the Institutional Review Board of Children’s Hospital of Chongqing Medical University (2022-177). Written informed consent to participate in this study was provided by the participants’ legal guardian/next of kin.

## Author contributions

JC and Z-ZZ were involved in the interpretation of the data, conceptualized the manuscript, and participated in the revisions. JC, Z-ZZ, and FL provided the financial support. JZ, TA, and KZ were involved in the acquisition of all the clinical data. Y-JZ, H-YG, and Q-QT were involved in the drafting of the manuscript and analyzed the data. B-YX, QW, and A-LH participated to the revisions. All authors approved the final version of the manuscript.
